# Co-inheritance of G6PD deficiency and 211 G to a variation of UGT1A1 in neonates with hyperbilirubinemia in eastern Guangdong

**DOI:** 10.1186/s12887-021-03010-6

**Published:** 2021-12-11

**Authors:** Jia-Xin Xu, Fen Lin, Zi-Kai Chen, Zhao-Yun Luo, Xiao-Fen Zhan, Jiao-Ren Wu, Yu-Bin Ma, Jian-Dong Li, Li-Ye Yang

**Affiliations:** 1 Central Laboratory, Chaozhou Central Hospital Affiliated to Southern Medical University, Chaozhou, 521021 Guangdong Province People’s Republic of China; 2 School of Food Engineering and Biotechnology, Hanshan Normal University, Chaozhou, 521021 Guangdong Province People’s Republic of China; 3 Department of Pediatrics, Chaozhou Central Hospital Affiliated to Southern Medical University, Chaozhou, 521021 Guangdong Province People’s Republic of China; 4 Precision Medical Center, People’s Hospital of Yangjiang Affiliated to Guangdong Medical University, No. 42 Dongshan Road, Yangjiang, 529500 Guangdong Province People’s Republic of China

**Keywords:** Neonatal hyperbilirubinemia, Glucose-6-phosphate dehydrogenase deficiency, Uridine diphosphate glucuronosyltransferase 1A1 (UGT1A1)

## Abstract

**Background:**

Glucose-6-phosphate dehydrogenase (G6PD) deficiency, which may manifest as neonatal hyperbilirubinemia, is the most prevalent erythrocytic enzyme-related disease in the world.

**Objective:**

To investigate the association between neonatal hyperbilirubinemia and co-inheritance of G6PD deficiency and 211 G to A variation of UGT1A1 in Chaozhou city of eastern Guangdong province, the effects of G6PD deficiency and UGT1A1 gene variant on the bilirubin level were determined in neonates with hyperbilirubinemia.

**Method:**

The activity of G6PD was assayed by an auto-bioanalyzer. PCR and flow-through hybridization were used to detect 14 common G6PD mutations in G6PD deficient neonates. 211 G to A variation of UGT1A1 was determined by PCR and sequencing. The data of neonatal bilirubin was collected and analyzed retrospectively.

**Results:**

Seventy four cases of the 882 hyperbilirubinemia neonates were G6PD deficiency (8.39%) while 12 cases of the 585 non-hyperbilirubinemia neonates (control group) were G6PD deficiency (2.05%). The rate of G6PD deficiency in the hyperbilirubinemia group was higher than that of the control group. Moreover, the peak bilirubinin of the G6PD-deficient group of hyperbilirubinemia neonates was 334.43 ± 79.27 μmol/L, higher than that of the normal G6PD group of hyperbilirubinemia neonates (300.30 ± 68.62 μmol/L). The most common genotypes of G6PD deficiency were *c.1376G > T* and *c.1388G > A*, and the peak bilirubin of neonates with these two variants were 312.60 ± 71.81 μmol/L and 367.88 ± 75.79 μmol/L, respectively. The bilirubin level of c.1388G > A was significantly higher than that of c.1376G > T. Among the 74 hyperbilirubinemia neonates with G6PD deficiency, 6 cases were 211 G to A homozygous mutation (bilirubin levels 369.55 ± 84.51 μmol/L), 27 cases were 211 G to A heterozygous mutation (bilirubin levels 341.50 ± 63.21 μmol/L), and 41 cases were wild genotypes (bilirubin levels 324.63 ± 57.52 μmol/L).

**Conclusion:**

The rate of G6PD deficiency in hyperbilirubinemia neonates was significantly higher than that of the non-hyperbilirubinemia neonates in Chaozhou. For the hyperbilirubinemia group, neonates with G6PD deficiency had a higher bilirubin level compared to those with normal G6PD. For hyperbilirubinemia neonates with G6PD deficiency, there was a declining trend of bilirubin levels among 211 G to A homozygous mutation, heterozygous mutation, and wild genotype, but there was no significance statistically among the three groups.

## Introduction

Neonatal hyperbilirubinemia can manifest as jaundice of skin, mucosa, and sclera, due to the excessive bilirubin in the neonates and the limited ability to deal with bilirubin originated from the decomposition of hemoglobin in red blood cells [1]. Bilirubin should be expelled from the body under normal condition, otherwise, its accumulation can induce cytotoxicity. Moreover, owing to the high renewal rate and short life span of red blood cells in neonates, neonatal bilirubin production is much faster than that of adults. On the other hand, the excretion of bilirubin in neonates is much slower than in adults [2, 3]. Therefore, bilirubin-related nuclear jaundice, nervous system damage, and even death often occur in neonates [4].

G6PD deficiency, the most common enzyme defect in humans, is one of the main risk factors of neonatal hyperbilirubinemia [5]. It is estimated that the global prevalence of G6PD deficiency is 4.9%, affecting about 330 million people, mostly from Africa, the Mediterranean, Southeast Asia, and Latin America [6, 7]. The incidence in China is higher in the Southern areas, including Hainan, Guangxi, Guangdong, Fujian, and Taiwan. To date, around 217 types of G6PD mutations have been reported worldwide while at least 31 mutations have been identified in the Chinese population [8, 9].

Non-conjugated bilirubin is bound to glucuronic acid to form conjugated bilirubin in the liver by uridine diphosphate glucuronosyltransferase (UGT) encoded by UGT1A1, and then excreted in vivo through a series of complex processes [10]. UGT1A1 gene mutations causing structural or functional defects of the enzyme can reduce the activity of UGT, resulting in increased serum bilirubin level. Among more than 100 mutation types of UGT1A1, the G to A variation at the nucleotide 211 of the UGT1A1 (UGT1A1*6, c.211G > A, p.Arg71Gly, rs4148323) is the most frequent mutation in the Asian cohort, contributes to hyperbilirubinemia in Asian neonates [11].

The purpose of this study was to explore the relationship between neonatal hyperbilirubinemia and G6PD deficiency and to investigate the effect of G6PD deficiency and UGT1A1 variant on bilirubin level of icteric neonates in Chaozhou, eastern Guangdong Province.

## Materials and methods

### Objects

The study was carried out in full-term neonates (gestational weeks between 37-42 weeks) admitted to Chaozhou Central Hospital Affiliated to Southern Medical University from January 2014 to August 2019. They were born from several hospitals of eastern Guangdong Province including Chaozhou Central Hospital, aged from 1 day to 28 days.

Hyperbilirubinemia group: All cases were full-term neonates with total serum bilirubin of more than 221 μmol/L, they were referred to our hospital for their yellow skin. Those cases with dehydration, ABO hemolysis, Rh hemolysis, serious infection septicemia, scalp hematoma, and other hemoglobin diseases were excluded from this analysis, and 882 cases were included in this study.

Control group: 585 cases. All were full-term with total serum bilirubin of lower than 21 μmol/L for age less than 14 days, and as for age beyond 14 days, total serum bilirubin was lower than 140 μmol/L, most of them were admitted into the hospital due to mild pneumonia.

This study was initially approved by the Ethics Committee of Chaozhou Central Hospital in 2011 (No. 2011021), and the second ethical approval was obtained in 2015 (No.2015001). As the clinical data were analyzed anonymously, and the blood samples in this study were obtained after the clinical diagnosis, a waiver of written consent was approved by the Ethics Committee of Chaozhou Central Hospital.

### Methods

#### Quantitative detection of G6PD enzyme activity

According to the National Inspection Operational Regulations, 1 mL solution (Co-Heath Beijing Laboratories Co., Ltd.) was added in a small cup, and then 20 μL of erythrocyte was accurately absorbed into the solution without the plasma layer. The activity of G6PD was detected by the rate method on Hitachi 7180 automatic biochemical analyzer (HITACHI, Japan), and the concentration of hemoglobin in hemolysis was detected by the HiCN method. This method can detect NADPH production in fixed time, which reflect G6PD enzyme activity in red blood cells. The reference range was 1300 U/L-3600 U/L, G6PD concentration lower than 1300 U/L was considered to be deficient, ≥1300 U/L was considered to be normal [12].

#### Extraction of genomic DNA

The DNA was extracted from the samples using the DNA prep kits (Chaozhou Hybribio Co., Ltd.) according to the instructions of the manufacturer. DNA concentration was determined by measuring the UV absorption at 260 nm with a spectrophotometer (NanoDrop One, Thermo Fisher Scientific Co., Ltd).

#### PCR and G6PD genotyping

PCR was performed in a 50 μL reaction system containing 46.4 μL PCR intermixture, 0.6 μL DNA polymerase, and 3 μL DNA (Chaozhou Hybribio Co., Ltd.) in an MJ Mini Personal Thermal Cycler (Bio-Rad Company) using the procedure as follows: pre-denaturation at 95 °C for 9 min; 40 cycles of denaturation at 95 °C for 30s, annealing at 56 °C for 30 s and extension at 72 °C for 1 min; and a final extension at 72 °C for 5 min.

Fourteen common mutations of G6PD in Chinese were detected by flow-through hybridization. This technique could simultaneously detect the 13 common G6PD gene mutations: *c.95A > G* (G6PD Gaohe), *c.392G > T* (G6PD Qing Yan), *c.487G > A* (G6PD Mahidol), *c.493A > G* (G6PD Taipei), *c.592G > T* (G6PD Coimbra), *c.871 G > A* (G6PD Viangchan), *c.1004C > T* (G6PD Fushan), *c.1024C > T* (G6PD Chinese-5), *c.1360C > T* (G6PD Union), *c.1376G > T* (G6PD Canton), *c.1387C > T* (G6PD Keelung), *c.1388G > A* (G6PD Kaiping)*, c.1381G > A* (G6PD Yunan) and one polymorphism *c.1311C > T* at the same time. The hybridization process was performed according to the manufacturer’s protocol (Chaozhou Hybribio Co., Ltd.) [13].

#### UGT1A1 genotyping

PCR was performed in a 25 μL reaction system containing 2 μL of DNA template, 12.5 μL 2 × Taq PCR Mix (Aidlab Biotechnologies Co., Ltd), 8.5 μL H_2_O, and 2 μL of primers (F5’-CATGCTGGGAAGATACTGTTG-3′, R5’-TTTGGTGAAGGCAGTTGATT-3′). The reaction was performed in an MJ Mini Personal Thermal Cycler (Bio-Rad Company) with an initial denaturation step at 95 °C for 3 min, followed by 35 cycles of denaturation at 94 °C for 30s, annealing at 58 °C for 30s, and extension at 72 °C for 50s, and a final extension step at 72 °C for 10 min. The PCR amplification products (775 bp) were sequenced by ABI 3730xL DNA Sequencer (PE Biosystems, CT, USA). The software Chromas was used to identify the subtype of gene mutation [14].

### Clinical data collection and statistical analysis

Sex, date of birth, weight, bilirubin level at admission, and date of hospital admission of all of the neonates were obtained from the clinical records.

Statistical analysis was performed by SPSS (version 19.0 statistical software SPSS Inc., Chicago, IL). The data from measurement was shown as mean ± standard deviation, and the data from counting was displayed as composition ratio or rate. The independent sample t test was used, *P* < 0.05 was considered statistically significant.

## Results

### Comparison of G6PD deficiency between neonates with and without hyperbilirubinemia

The numbers of neonates with G6PD deficiency in the hyperbilirubinemia and control groups were shown in Table 1. Seventy four cases (8.36%, 67 males and 7 females) were G6PD deficient in 882 neonates with hyperbilirubinemia.12 cases (2.05%, 10 males and 2 females) were G6PD deficient among 585 controls. The proportion of G6PD deficiency in the hyperbilirubinemia group was significantly higher than that of the control group (OR = 4.38, *P* < 0.01). Feeding patterns in the group of hyperbilirubinemia were summarized in Table 2.

**Table 1:** Comparison of G6PD deficiency between the hyperbilirubinemia group and the control group

	G6PD deficiency (n/proportion)	normal G6PD (n/ proportion)	*P* value
Hyperbilirubinemia group	74 / 8.39%	808 / 91.61%	<0.001
Control group	12 / 2.05%	573 / 97.95%

**Table 2:** Feeding patterns in the group of hyperbilirubinemia

		Breastfeeding	Breast and formula	Formula	Unknown
Normal G6PD		204	239	203	164
G6PD deficiency	UGT1A1c.211A/A Homozygote	1	2	2	1
	UGT1A1 c.211G/A Heterozygote	8	4	11	4
	UGT1A1 c.211G/G Wild type	8	11	15	7
	Total	17	17	28	12

### Comparison of the bilirubin level between the G6PD-deficient group and the normal group of neonates with hyperbilirubinemia

**Fig. 1:**
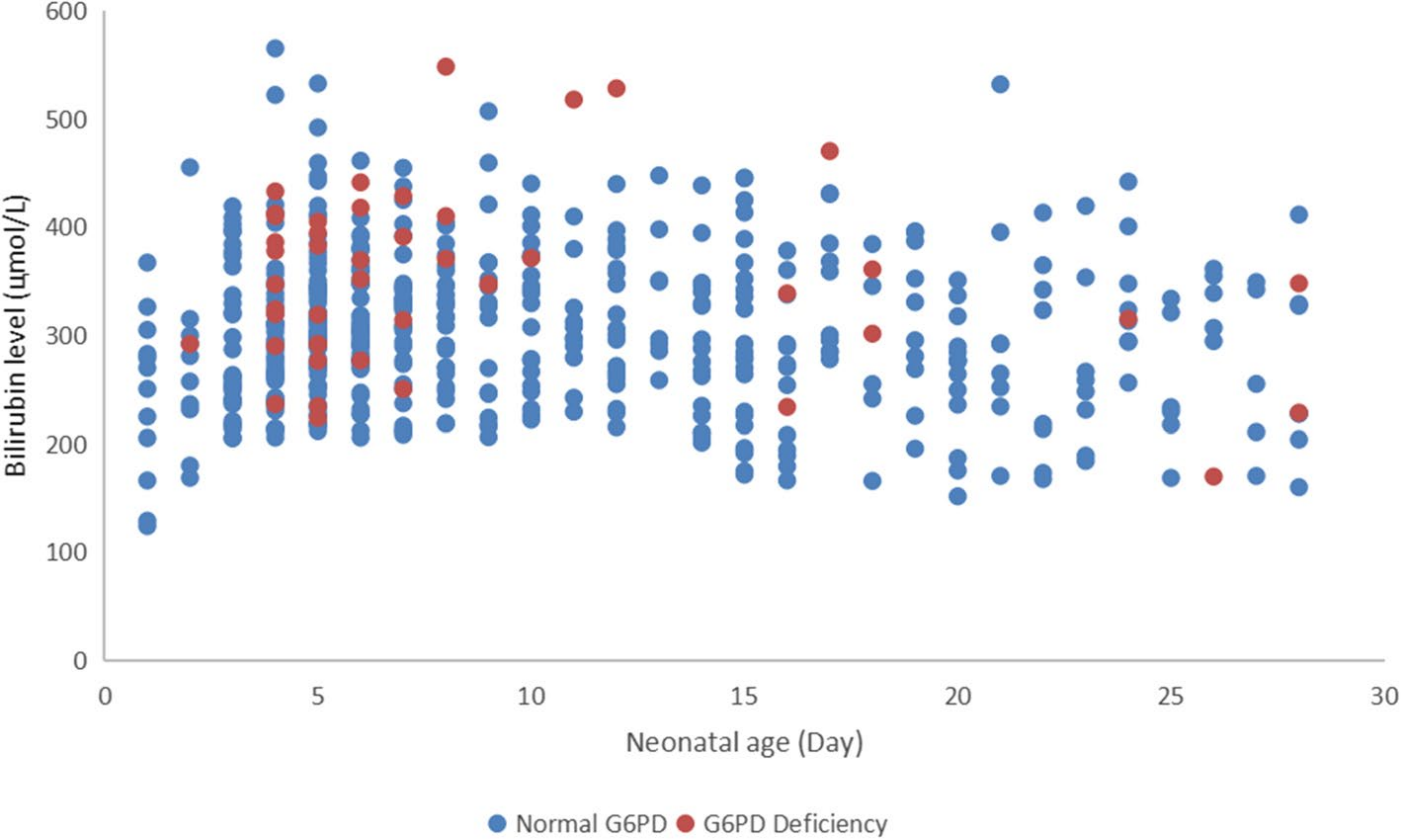
The comparison of bilirubin levels in G6PD-deficient and G6PD-normal jaundiced neonates

The bilirubin level at admission and postnatal days of the 882 jaundiced neonates were shown in Fig. 1. In 808 cases of jaundice with normal G6PD, the admission level of total bilirubin was 300.30 ± 68.62 μmol/L, and the admission bilirubin level in 74 cases with G6PD deficiency was 334.43 ± 79.27 μmol/L, which was markedly higher than that of the former (*P* < 0.01).

### The mutations of the G6PD gene

**Fig. 2:**
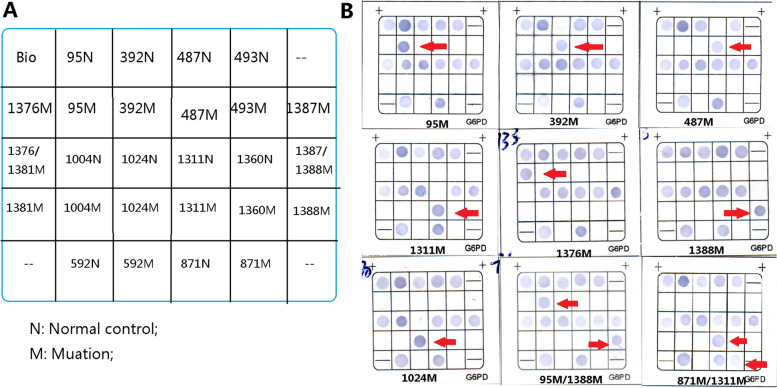
**A** The location of the probes dotted in the gene chip used for the flow-through hybridization. Location of wild-type was denoted as N. Location of mutant probes was denoted as M. **B** Part of hybridization results of G6PD gene chip. 95 M, 392 M, 487 M, 1311 M, 1376 M, 1388 M, 1024 M, 95 M/1388 M, and 871 M/1311 M were positive on the G6PD gene chip

The G6PD genotypes of 74 hyperbilirubinemia cases with G6PD-deficient were determined by flow-through hybridization. All probes localization on the membrane was shown in Fig. 2A while the hybridization results of the G6PD gene chip were shown in Fig. 2B.

Gene mutations were found in 69 cases, mainly were *c.1376G > T* (34/74, 45.95%) and *c.1388G > A* (26/74, 35.14%), followed by *c.392G > T* (2/74, 2.70%), *c.95A > G* (2/74, 2.70%), *c.871G > A/c.1311C > T* (2/74, 2.7%), and *c.1024C > T* (1/74, 1.35%). Single*c.1311C > T* polymorphism was found in one case (1/74, 1.35%). The mean values of the admission bilirubin of *c.1376G > T* and *c.1388G > A* were 312.60 ± 71.81 μmol/L and 367.88 ± 75.79 μmol/L, respectively. The bilirubin level of neonates with *c.1388G > A* was significantly higher than that of neonates with *c.1376G > T* (*P* < 0.05). Moreover, there were two kinds of double heterozygote, i.e. *c.1311C > T/c.871 G > A* (2/74, 2.7%) and *c.1388G > A/c.95A > G* (1/74, 1.35%). No mutation was detected in the remaining 4 cases.

The distribution of sex, bilirubin levels, and days of the birth of the 74 jaundiced neonates with G6PD deficiency in different types of gene mutation were shown in Table 3. Interestingly, different types of G6PD gene mutation tended to appear at different postnatal days with the peak bilirubin level. For example, *c.1388G > A* mutation made the highest proportion (6/9, 66.67%) in 1-3 days, followed by *c.1376G > T*. In 4-5 days, the *c.1376G > T* mutation was the primary type (12/22, 54.55%), followed by *c.1388G > A*. The G6PD gene mutation was dominated by *c.1376G > T* (19/43, 44.19%) beyond 5 days.

**Table 3:** The genotypes of G6PD deficiency and bilirubin level in jaundiced neonates

Type of mutation	M* (n)	F* (n)	Proportion (%)	Total bilirubin (μmol/L)	Cases number in different postnatal days								
					<1d	<2d	<3d	<4d	<5d	<6d	<7d	7-14d	14-28d
*c.1376G > T*(Canton)	31	3	45.95	312.60 ± 71.81	0	1	2	7	5	7	2	5	5
*c.1388G > A*(Kaiping)	25	1	35.14	367.88 ± 75.79	1	1	4	4	3	3	1	6	3
*c.95A > G*(Gaohe)	2	0	2.70	428.95 ± 168.22	0	0	0	0	0	1	0	1	0
*c.392G > T*(Qing Yan)	2	0	2.70	264.64 ± 93.52	0	0	0	1	0	0	0	1	0
*c.487G > A*(Mahidol)	1	0	1.35	332	0	0	0	0	0	1	0	0	0
*c.1024C > T*(Chinese-5)	1	0	1.35	306	0	0	0	0	1	0	0	0	0
*c.1311C > T*	1	0	1.35	318.3	0	0	0	0	0	0	0	0	1
*c.1311C > T/* *c.871 G > A* (Viangchan)	1	1	2.70	418.6	0	0	0	0	0	1	0	0	1
*c.1388G > A/* *c.95A > G*	0	1	1.35	280.1	0	0	0	0	0	0	0	0	1
Unidentified	3	1	5.41	319.65 ± 69.55	0	0	0	0	1	0	1	2	0
Total	67	7	100	334.43 ± 79.27	1	2	6	12	10	13	4	15	11

M* Male, F* Female

In the 12 neonates with G6PD deficiency of normal bilirubin level (control group), G6PD gene mutations were identified in 9 cases, including *c.1376G > T* (5 cases), *c.1388G > A* (2 cases), *c .1024C > T* (1 case), and *c.95A > G* (1 case).

Bilirubin encephalopathy occurred in three jaundiced newborns with G6PD deficiency in this study. The mutant genotypes of the 3 cases were *c.1376G > T*, *c. 1388G > A,* and *c. 95A > G*, respectively, and the peak bilirubin level was 390.8 μmol/L (age: 7 days), 527.89 μmol/L (age: 12 days), and 547.9 μmol/L (age: 8 days), respectively.

### Comparison of serum bilirubin levels of different UGT1A1 genotypes in jaundiced neonates with G6PD deficiency

In 74 G6PD-deficient cases with hyperbilirubinemia, there were 6 cases of homozygous mutation of c.211G *>* A, 27 cases of heterozygous mutation, and 41 cases of wild type. The serum bilirubin levels were 369.55 ± 84.51 μmol/L, 341.50 ± 63.21 μmol/L, and 324.63 ± 57.52 μmol/L, respectively. For hyperbilirubinemia neonates with G6PD deficiency, there was a declining trend of bilirubin levels among 211 G to A homozygous mutation, heterozygous mutation, and wild genotype, but there was no significant difference statistically for bilirubin level among the three groups (*P* > 0.05).

There were 808 cases of hyperbilirubinemia without G6PD deficiency, 49 cases were randomly selected for genotyping of UGT1A1, 22 cases were wild type, 21 cases were c.211G *>* A heterozygous mutation, 6 cases were homozygous mutation of c.211A/A. There was no significance of c.211 alleles distribution between the G6PD deficiency hyperbilirubinemia group and normal G6PD hyperbilirubinemia group (*P* = 0.137).

585 neonates with normal bilirubin levels were set as control group in this study, we did not performed UGT1A1 variants analysis for all 585 cases, only 65 cases of the control group were analyzed in our previous report [15], 51 cases were wild genotype of c.211G/G, 13 cases were heterozygotes for c.211G > A (p.Arg71Gly), 1 case was homozygote of c.211A/A. There was significance of c.211 alleles distribution between the G6PD deficiency hyperbilirubinemia group and normal bilirubin control group statistically (*P* = 0.001).

## Discussion

G6PD deficiency is a common hereditary blood disease in southern China. It is prevalent in Chaozhou, eastern Guangdong province. The rate of G6PD deficiency in Chaozhou neonates was 2.68% according to our previous screening [16]. In this study, we mainly focused on the neonates with hyperbilirubinemia and found that the incidence of G6PD deficiency in this cohort was 8.39% (74/882), which was 3-fold higher than that of neonates without hyperbilirubinemia (2.05%, 12/585). Thus, G6PD deficiency was a risk factor for neonatal hyperbilirubinemia. Importantly, for the 74 hyperbilirubinemia neonates with G6PD deficiency, 67 cases were males (90.5%) while only 7 cases were females (9.5%). This was likely attributed to the G6PD gene location on the X chromosome and the resulting sex linkage. Female heterozygotes may be difficult to diagnose because of X-chromosome mosaicism leading to a partial deficiency that will not be detected reliably with screening tests [17].

In the neonates with hyperbilirubinemia, the bilirubin level of 74 cases of G6PD deficiency was 334.43 ± 79.27 μmol/L, which was markedly higher than that of neonates with normal G6PD (300.30 ± 68.62 μmol/L). Furthermore, neonatal hyperbilirubinemia combining with the *c.1388G > A* mutation resulted in earlier jaundice and higher bilirubin level. Therefore, we should be alert to the G6PD deficiency when the neonates suffered from hyperbilirubinemia during clinical practice. In Guangdong province of China, G6PD screening was a routine procedure for newborns at birth, if G6PD deficiency was identified in neonates, their parents would be informed and G6PD deficiency associated disorders such as neonatal jaundice was especially cautioned, G6PD deficiency neonates should receive transcutaneous bilirubin measurement regularly. In this study, bilirubin encephalopathy occurred in three jaundiced newborns with G6PD deficiency. The pathogenesis of significant neonatal hyperbilirubinemia was often multifactorial, which might encompass several environmental and genetic contributors [3]. Therefore, the genetic detection was not enough for preventing severe hyperbilirubinemia. For those neonates with jaundice contributors, routinely transcutaneous bilirubin measurement was recommended to avoid severe hyperbilirubinemia and bilirubin encephalopathy.

Lin et al. have shown that *c.1376G > T*, *c.1388G > A*, and *c.1024C > T* were the most common mutations of G6PD in Chaozhou, accounting for 75.6% of G6PD-deficient cases [18]. Similarly, we found that the majority of G6PD variants were *c.1376G > T* (34/74, 45.95%) and *c.1388G > A* (26/74, 35.14%), and there was only one case with *c.1024C > T*. The diagnostic criteria for severe hyperbilirubinemia was bilirubin level over 342 μmol/L [19]. In our study, the mean peak bilirubin of neonates with *c.1388G > A* was 367.88 ± 75.79 μmol/L, indicating that *c.1388G > A* lead to severe neonatal hyperbilirubinemia easily. This result was consistent with a previous study in Malaysian Chinese [20]. Theoretically, *c.1388G > A* causes substitution of the Arg^453^ of G6PD to a histidine, which is close to the NADP binding region and affects the integration and function of G6P and NADP [21]. Therefore, *c.1388G > A* often causes severe clinical symptoms. Due to the limited number of cases studied, the relationship between bilirubin level and neonatal hyperbilirubinemia could not be determined in other types of G6PD mutation.

Most patients with G6PD deficiency have only one mutation, while a few patients have multiple mutations. In our study, 1 case (1 female) showed compound mutation of c.1388G > A/c.95A > G, and the bilirubin in this neonate was 280.1 μmol/L. Whether the level of bilirubin is related to the types of multiple mutations remains to be further studied.

During bilirubin metabolism process, gene mutation can cause bilirubin conjugation blockage and abnormal accumulation in vivo, resulting in neonatal hyperbilirubinemia. Whether the cumulative mutations of G6PD and UGT1A1 are related to more severe neonatal hyperbilirubinemia requires further study. TATA mutation of UGT1A1 promoter coupled with G6PD deficiency was indicated as an important factor for neonatal hyperbilirubinemia in Nigeria [22]. Yang et al. showed that G6PD deficiency combined with UGT1A1c.211G > A homozygous mutation can increase the risk of severe hyperbilirubinemia [15]. In the current study of hyperbilirubinemia group with G6PD deficiency, we showed that 6 cases were UGT1A1 *c.211G > A* homozygous mutation, 27 cases were heterozygous, and 41 cases were wild. Although the jaundice tended to be more severe with the increase of mutations, the difference in bilirubin levels among them was not statistically significant. It may be due to the limited number of cases and requires larger enrollment of neonates in further study.

Unexpectedly, common G6PD mutations were not detected in 4 G6PD deficient neonates with hyperbilirubinemia (3 males, 1 female) and 3 G6PD deficient neonates without hyperbilirubinemia (3 males). This may be due to that the kit for detection of G6PD mutation can only detect 14 common mutations in the Chinese population, and there may be unusual mutations in these cases, which need to be verified by gene sequencing. Moreover, mutations of other genes, such as heme oxygenase-1 (HO-1), biliverdin reductase A (BLVRA), and solute carrier organic anion transporter family member 1B1 (SLCO1B1) could also affect the serum bilirubin [23].

## Conclusion

G6PD deficiency has important clinical significance for neonatal hyperbilirubinemia. We proposed that the neonates with hyperbilirubinemia should be screened for G6PD activity actively and timely. This can help to analyze the etiology and characteristics of neonatal jaundice and to prevent serious kernicterus or even death with prompt and effective treatment. If possible, universal screening for G6PD deficiency at birth was recommended, percutaneous bilirubin in newborns with G6PD deficiency should be monitored regularly to avoid severe jaundice happening.

## Data Availability

The datasets used and/or analysed during the current study are available from the corresponding author on reasonable request.

## Abbreviations

G6PD: Glucose-6-phosphate dehydrogenase deficiency
UGT1A1: Uridinediphosphateglucuronosyltransferase 1A1
PCR: Polymerase chain reaction
HO-1: Oxygenase-1
BLVRA: Biliverdin reductase A
SLCO1B1: Solute carrier organic anion transporter family member 1B1
